# Nature of the liver volume depending on the gender and age assessing volumetry from a reconstruction of the computed tomography

**DOI:** 10.1371/journal.pone.0261094

**Published:** 2021-12-08

**Authors:** Kohei Harada, Tomohiro Ishinuki, Yoshiya Ohashi, Takeo Tanaka, Ayaka Chiba, Kanako Numasawa, Tatsuya Imai, Shun Hayasaka, Takahito Tsugiki, Koji Miyanishi, Minoru Nagayama, Ichiro Takemasa, Junji Kato, Toru Mizuguchi

**Affiliations:** 1 Division of Radiology, Sapporo Medical University Hospital, Sapporo, Hokkaido, Japan; 2 Postgraduate School of Health Science and Medicine, Sapporo Medical University, Sapporo, Hokkaido, Japan; 3 Department of Medical Oncology, Sapporo Medical University, Sapporo, Hokkaido, Japan; 4 Departments of Surgery, Surgical Science and Oncology, Sapporo Medical University, Sapporo, Hokkaido, Japan; University of Tsukuba, JAPAN

## Abstract

Although the liver is a regenerating organ, excessive loss of liver volume (LV) can cause fatal liver failure. It is unclear whether LV is correlated with age; however, it is known that liver function decreases with age. In addition, the gender-related role of LV remains unclear. This study aimed to investigate the changes in LV by age and gender. Between January and December 2018, 374 consecutive patients who underwent abdominal multidetector computed tomography (MDCT) for any abdominal examinations were enrolled. LV was evaluated using MDCT. The relationship between the LV and body mass index (BMI), body surface area (BSA), age, and gender was investigated. The modified LV (mLV) was calculated by a formula measured LV × 1.5/BSA. LV correlated to BSA more than to BMI in both the males (R: 0.559 vs. 0.416) and females (R: 0.479 vs. 0.300) in our study. Age was negatively correlated to LV and BSA, and correlated to LV more than to BSA in males (R: 0.546 vs. 0.393) and females (R: 0.506 vs. 0.385). In addition, the absolute slope between age and LV in the males was higher than that in the females (14.1 vs. 10.2, respectively). Furthermore, the absolute slope of age and mLV in the males was slightly higher than in the females (9.1 vs. 7.3, respectively). In conclusion, LV in the normal liver is correlated to age rather than the one in the diseased liver. Liver volume in the males decreased more with age than LV in the females.

## Introduction

The liver can regenerate to recover the original mass once it is removed surgically [1, 2]. In clinical settings, liver resection is required to remove liver tumors or to donate for living organ transplantation [3, 4]. Excess loss of liver volume (LV) due to oversized hepatectomy can lead to fatal liver failure because the liver regenerates insufficiently [5–7]. Due to the fact that the liver cannot regenerate in some instances, considerable research has been conducted in an effort to overcome the limitation of liver regeneration.

Studies have investigated liver regeneration mechanisms in both in vivo and in vitro models [8, 9]. However, some hepatocytes could regenerate as stem cells; 90% volume loss causes liver failure [7]. In living liver transplantation, up to 70% of the liver can be removed from the donor organ, and a minimum of 30% LV should be preserved for donor safety [10, 11]. Posthepatectomy liver failure (PHLF) incidence is reportedly 8% to 12% [12–15]. The regenerative ability differs by gender and aging. Bachellier et al. reported that PHLF in male patients was higher than that in female patients [16]. It has also been reported that hepatectomy in elderly patients >70 years old has a high incidence of mortality [17]. However, the exact difference between the genders along with aging, is not well understood.

LV is affected by various factors such as body physique, blood flow, hormones, and liver damage. LV in chronic hepatitis and liver cirrhosis is lower than that in the normal liver [18]. Irreversible liver failure also occurs in fulminant hepatitis, which causes rapid liver atrophy. Specifically, the progression of liver disease affects LV. Conversely, body physique affects LV. The body’s physique increases with age due to decreased basic metabolism by the end of middle age of approximately 60 years. However, physique decreases afterward, resulting in frailty in the elderly. In addition, body physique between the genders differs from hormonal and metabolic characteristics. However, the relationships between aging and LV are not well known, and the underlying mechanisms of how body physic and metabolism affect LV is also unclear.

Traditionally, personal LV was estimated from body height and weight [19–23]. Accurate liver volumetry measures are now possible using multidetector-raw computed tomography (MDCT). Although attempts have been made with ultrasound and MRI [24, 25], MDCT is significantly superior in spatial resolution and reproducibility. Clarifying the relationship between LV and liver function in hepatectomy is important to avoid PHLF. Various formulas for estimating standard LV have been reported [19, 22, 26–28]. However, few of them considered the effect of gender, and none of them addressed the effect of aging.

This study aimed to investigate the changes in LV by aging and gender differences. We used modified LV (mLV) adjusted by the constant body surface area to avoid physical bias. This study showed a clear difference between the genders along with the aging effect using the latest radiological technology.

## Materials and methods

### Patient selection and scan parameters

Between January 2018 and December 2018, 374 consecutive patients who underwent abdominal dynamic studies using MDCT were enrolled in this study. Patients with previous malignancies, past catheterization, or past hepatectomy were excluded from this series. There was no duplicated participant.

Clinical laboratory tests, including albumin (ALB), bilirubin (Bil), aspartate aminotransferase (AST), alanine aminotransferase (ALT), and platelet (PLT), were evaluated before computed tomography examination.

LV was evaluated by 80-law MDCT (Aquilion PRIME; Canon Medical Systems, Tokyo, Japan). The images were obtained by three-phase methods, including the late arterial phase, portal venous phase, and late phase. Nonionic contrast material (Iomeron; Eisai, Tokyo, Japan) was administered intravenously for 30 seconds utilizing a power injector (Dual Shot GX7; Nemoto Kyorindo, Tokyo, Japan). The scanning delays of the late arterial phase were individually determined using a bolus-tracking technique. A circular cursor was placed on the aorta, and the late arterial phase scan was started 15 seconds after a 200 Hounsfield unit threshold was reached. The portal venous phase was acquired 30 seconds after the late arterial phase scan, and the late phase was acquired 180 seconds after the start of administration of the contrast material. A ZIO STATION 2 (Ziosoft Inc., Tokyo, Japan) was used to calculate the LV. The images of the portal venous phase were used for volumetry.

**Fig 1:**
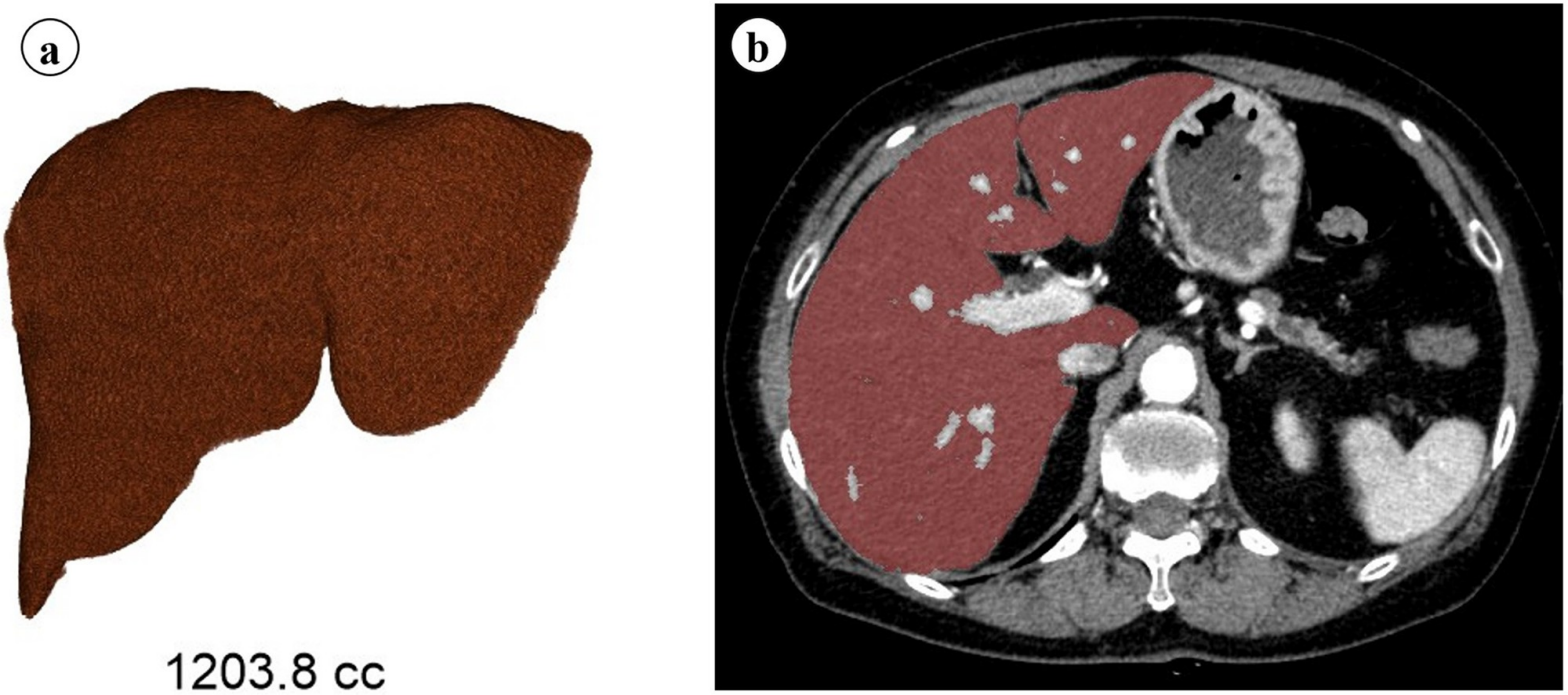
CT volumetry of the liver in the portal venous phase. The image on the left shows volumetry whereas the image on the right shows a mask image.

Vascular vessels such as the portal vein, hepatic vein, and inferior vena cava were excluded from volumetry (Fig 1). Additionally, benign tumors, such as liver cysts and hemangiomas, were excluded from volumetric measurements.

The relationship between the measured LV and BMI, BSA, age, and gender was investigated. For BSA, the formula of DuBois (BSA [m^2^] = 0.007184 × height [cm] ^0.725^ × weight [kg] ^0.425^) was used [29]. The mLV was calculated by a formula measuring LV×1.5/BSA, based on a BSA of 1.5 m^2^ for all patients.

This study was conducted according to the ethical guidelines of the Declaration of Helsinki. The Internal Review Board of Sapporo Medical University approved this study (ID: 302–195, approval date: February 14, 2019). Written informed consent was obtained from all participants prior to registry.

### Statistical analysis

Patient demographics and perioperative laboratory tests were extracted from the database, and differences between the groups were compared using the chi-square test followed by a post-hoc 2 × 2 Fisher’s exact test. The unpaired t test was used for comparisons between the males and the females. The relationships among the various clinical parameters were evaluated using Spearman’s rank correlation coefficient. All calculations were performed using the JMP 15.0 software program (SAS Institute Inc., London, ON, CAN). All results were expressed as the mean, together with minimum and maximum levels. *P* < 0.05 was considered statistically significant.

## Results and discussion

Table 1 shows the clinical background of the patients in this study. Characteristics including BMI, BSA, LV, mLV, AST, ALT, and PLT are significantly different between the males and the females. Conversely, the number of patients, age, ALB, and Bil were not significantly different between the groups. Table 2 shows the etiologies of the patients. In the males, 55 patients (28.6%) comprised the diseased liver group, and 137 patients (71.4%) comprised the normal liver group. In the females 44 patients (24.2%) comprised the diseased liver group, and 138 patients (75.8%) comprised the normal liver group. The etiologies between the groups were not significantly different.

**Table 1:** Comparison of characteristics between male and female study participants.

	Male	Female	P value
n	192	182	-
Age (years)	66.7 ± 12.3	66.7 ± 12.3	0.9981
BMI	22.74 ± 3.64	21.71 ± 3.13	0.0012
BSA (m^2^)	1.69 ± 0.16	1.47 ± 0.13	<0.0001
LV (mL)	1230.3 ± 316.9	995.3 ± 247.3	<0.0001
mLV (mL)	1085.1 ± 234.9	1010.2 ± 213.7	0.0043
ALB (g/dL)	3.77 ± 0.59	3.81 ± 0.53	0.8271
Bil (mg/dL)	1.02 ± 2.22	0.83 ± 1.20	0.2215
AST (IU/L)	44.9 ± 91.3	32.1 ± 46.1	0.0116
ALT (IU/L)	43.6 ± 85.3	32.7 ± 71.5	0.0096
PLT (10^4^/μL)	20.29 ± 9.01	22.47 ± 7.95	0.0026

BMI: body mass index, BSA: body surface area, LV: liver volume, mLV: modified liver volume. ALB: albumin, Bil: bilirubin, AST: aspartate aminotransferase, ALT: alanine aminotransferase, PLT: platelet. Data represent the mean + standard deviation.

**Table 2:** Clinical backgrounds of patients with underlying disease separated by gender.

	DL		Normal		DL		Normal
Males n	55		137	Female n	44		138
HB hepatitis	11	Esophago-Gastric cancer	8	HB hepatitis	12	Esophago-Gastric cancer	4
HC hepatitis	6	Colorectal cancer	17	HC hepatitis	5	Colorectal cancer	9
Other hepatitis	14	Retroperitoneal tumor	4	Other hepatitis	13	Retroperitoneal tumor	8
HCC	6	Renal cancer	9	HCC	2	Renal cancer	9
Bile duct disease	18	Pancreatitis	11	Bile duct disease	12	Pancreatitis	9
		Pancreatic tumor	61			Pancreatic tumor	70
		Metastatic tumor	5			Metastatic tumor	3
		Gallbladder stone	3			Gallbladder stone	3
		Gallbladder cancer	5			Gallbladder cancer	3
		Other tumors	14			Other tumors	20

DL: diseased liver, HCC: hepatocellular carcinoma.

**Fig 2:**
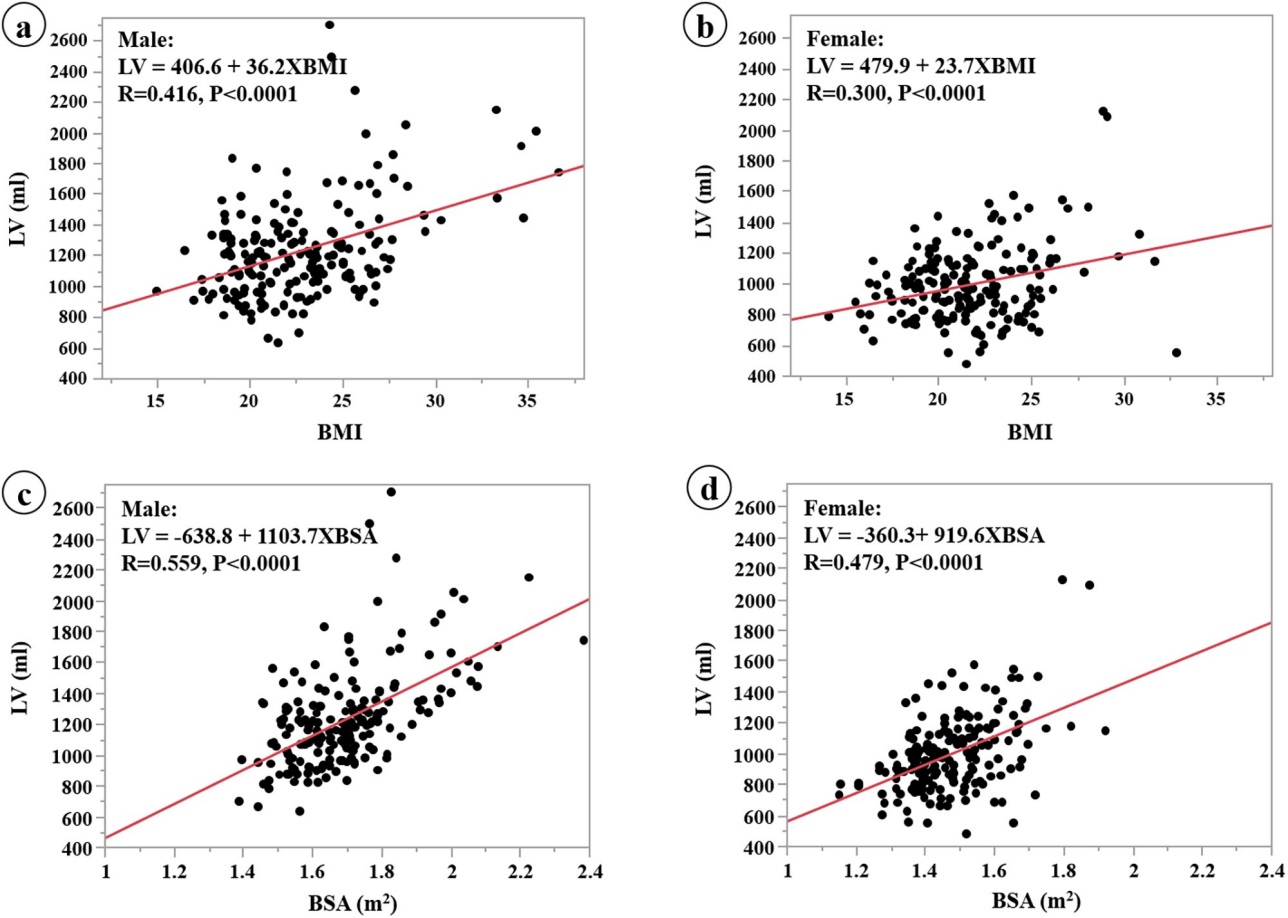
Linear regression analysis of LV and BMI by gender (a, b). Linear regression analysis of LV and BSA by gender (c, d). LV is more strongly correlated with BSA than BMI, with the correlation being stronger in males.

Scatter plots of the linear regression analysis between LV and BMI for the males and the females are shown in Fig 2a and 2b. Scatter plots between LV and BSA for the males and the females are shown in Fig 2c and 2d. LV is significantly correlated with BSA and BMI in the males and the females. The correlation (R) between LV and BSA is stronger than between LV and BMI in males and females (0.559 vs. 0.416 and 0.479 vs. 0.300, respectively). Also, the slope between LV and BSA in the males is higher than in the females (1103.7 vs. 919.6, respectively).

**Fig 3:**
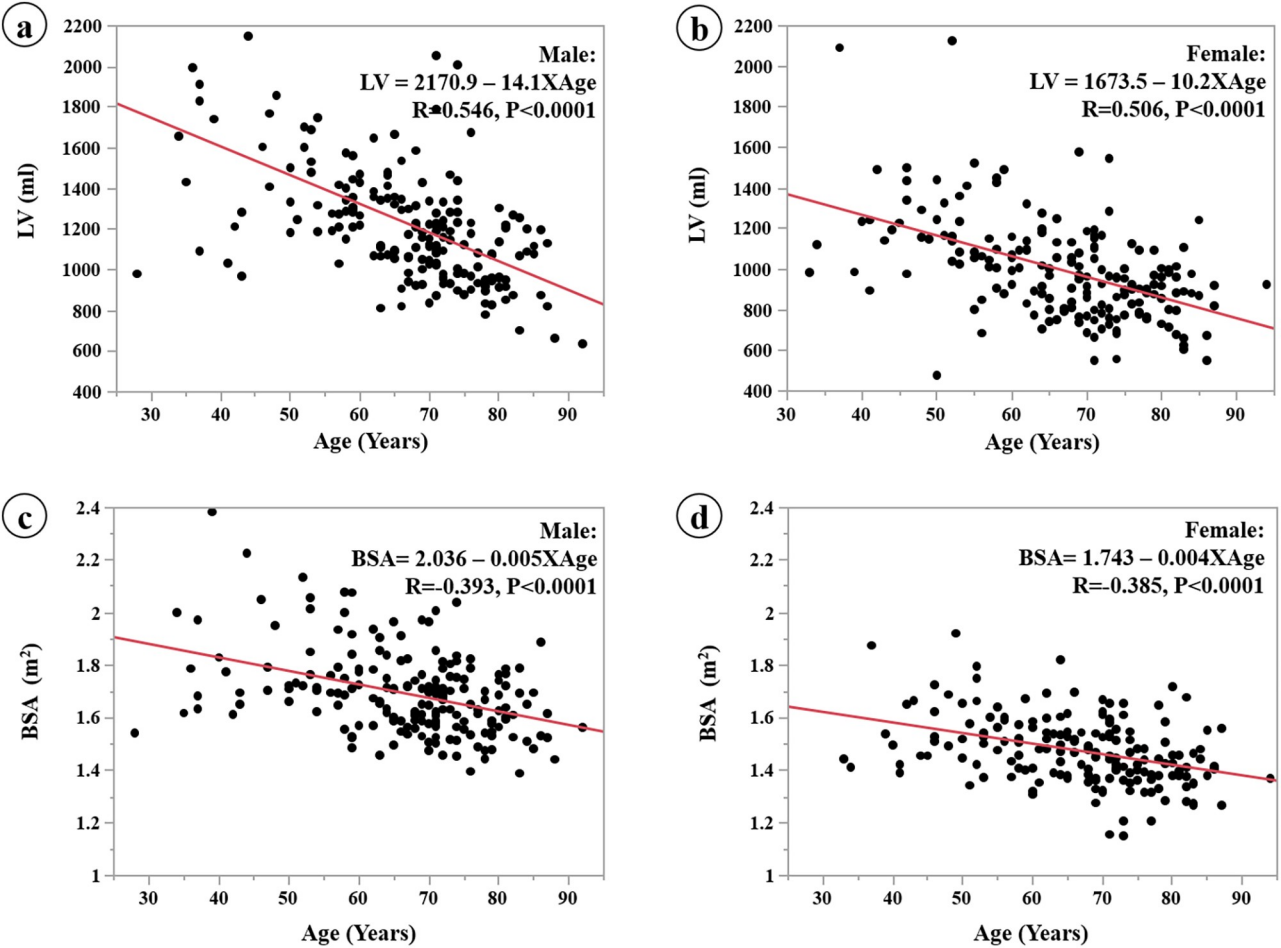
Linear regression analysis of LV and age by gender (a, b). Linear regression analysis of BSA and age by gender (c, d). The correlation between LV and age is stronger than the correlation between LV and BSA, and the slope is greater in the males.

Scatter plots of the linear regression analysis between LV and age for the males and the females are shown in Fig 3a and 3b. Scatter plots between BSA and age for males and for females are shown in Fig 3c and 3d. Age was negatively correlated with LV and BSA. The correlation (R) between LV and age is stronger than between BSA and age in the males and females (0.546 vs. 0.393, and 0.506 vs. 0.385, respectively). Furthermore, the correlation between LV and age in the males is higher than that in the females (14.1 vs. 10.2, respectively).

**Fig 4:**
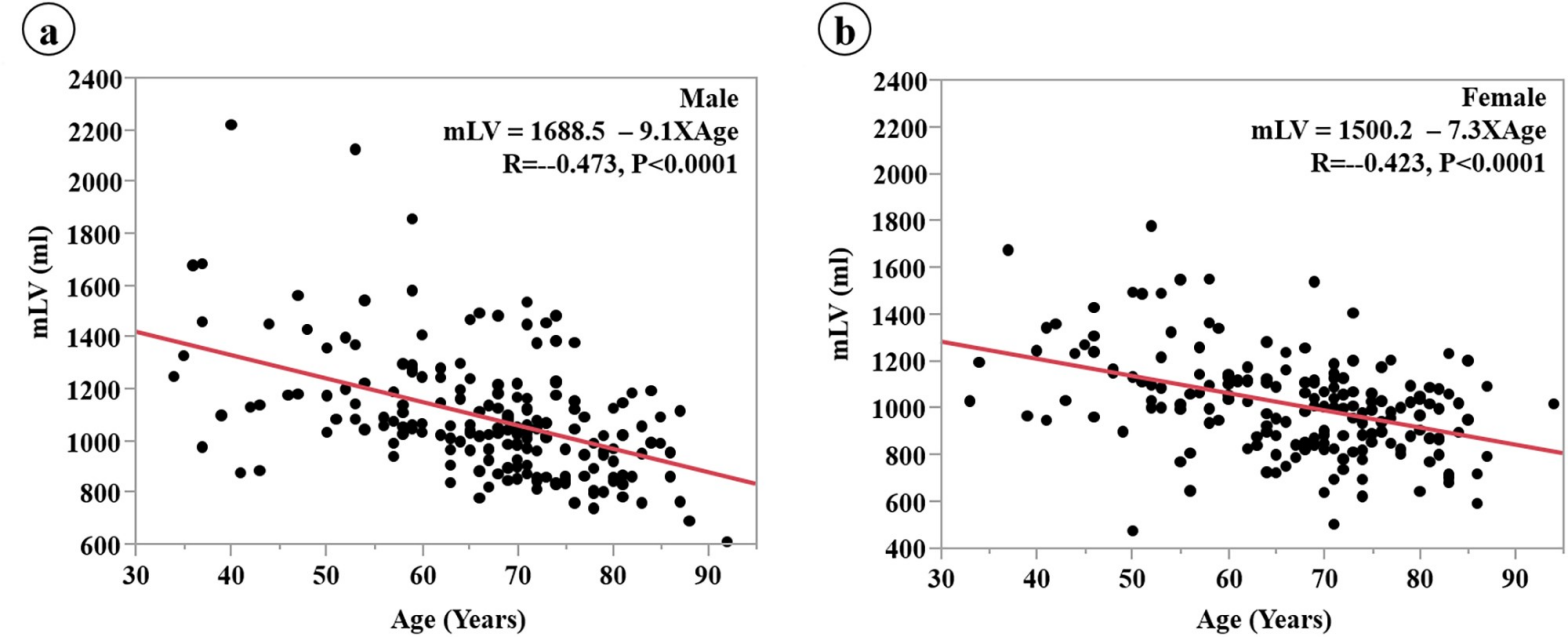
Linear regression of the relationship between mLV and age in participants separated by gender when the BSA of the target group is assumed to be 1.5 m^2^. The correlation between mLV and age is similar for males and females; however, the slope is slightly greater for males.

Scatter plots of the linear regression analysis between mLV and age for the males and the females are shown in Fig 4a and 4b. The correlation (R) of the males for LV and BSA was comparable with that of the females (0.473 vs. 0.423, respectively). However, the slope of the correlation between mLV and age in the males is slightly higher than that in the females (9.1 vs. 7.3, respectively).

**Fig 5:**
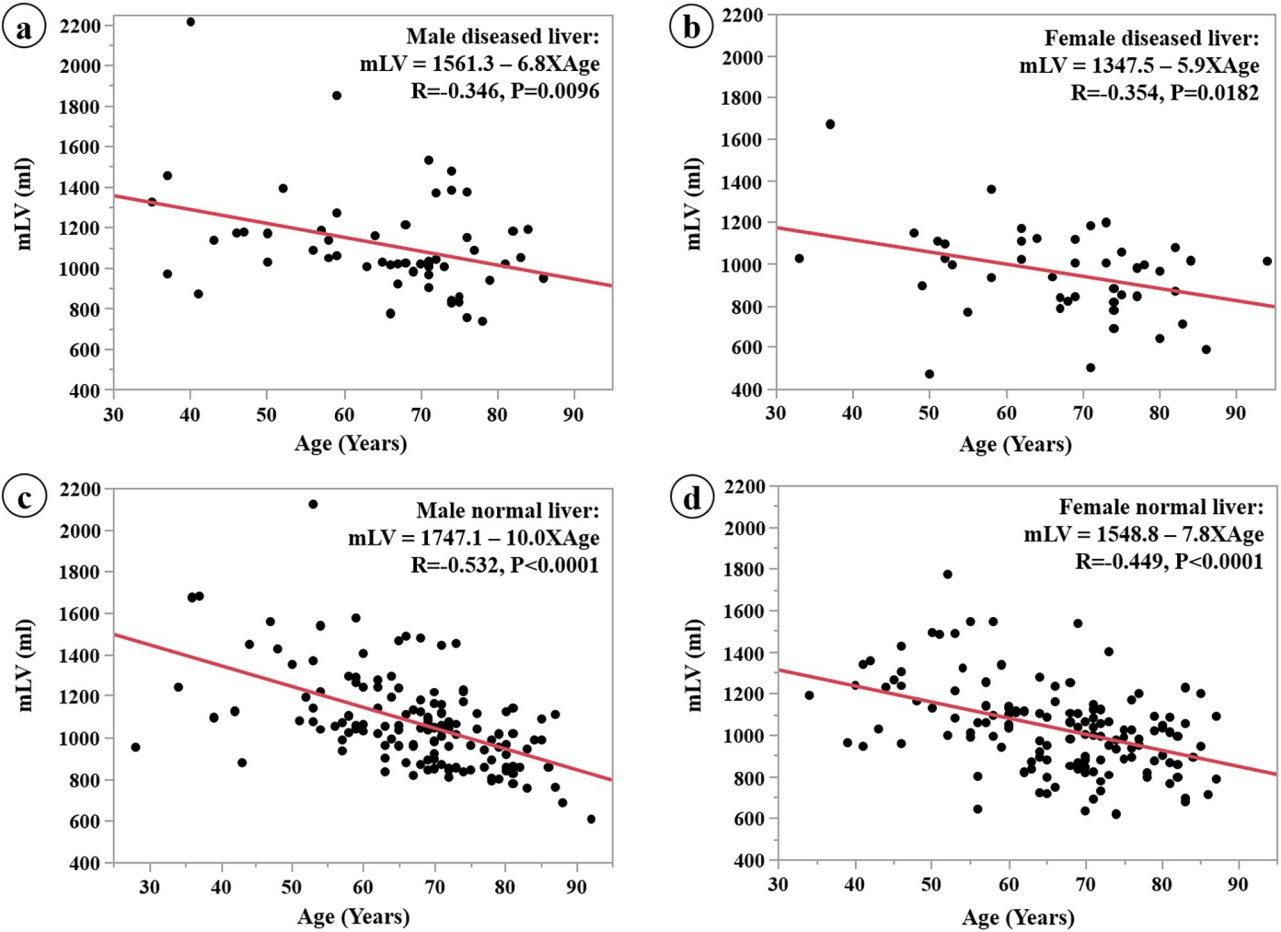
Linear regression of mLV and age in diseased and normal liver in males (a, c) and females (b, d). The correlation between mLV and age in the diseased liver group is weaker than the correlation between mLV and age in the normal liver group. The slope in the normal group is slightly greater in males than in females.

Scatter plots of the linear regression analysis between mLV and age for the males and females in the diseased liver group are shown in Fig 5a and 5b. Scatter plots between mLV and age for the males and females in the normal liver group are shown in Fig 5c and 5d. The correlation (R) in the diseased liver group between mLV and age was weaker than that in the normal liver group for the males and females (0.346 vs. 0.532, and 0.354 vs. 0.449, respectively). Furthermore, the slope of the correlation between mLV and age for the normal liver group in the males is slightly higher than that in the females (10.0 vs. 7.8, respectively).

Characteristics including BMI, BSA, LV, mLV, AST, ALT, and PLT differed significantly between males and females. However, the number of patients, age, ALB, and Bil did not differ significantly between the groups. Table 2 shows the etiologies of the patients. Among males, the diseased liver group comprised 55 patients (28.6%) and normal liver group comprised 137 patients (71.4%). Among females, the diseased liver group comprised 44 patients (24.2%) and normal liver group comprised 138 patients (75.8%). The etiologies between the groups were not significantly different.

Table 3 shows the correlation between LV and biochemical blood test data in males with normal and diseased liver. Bilirubin correlated with LV in the male diseased liver group (P = 0.004). Table 4 shows the correlation between LV and biochemical blood test data in females with normal and diseased liver. Platelets correlated with LV regardless of liver state in females (P < 0.001, P = 0.039). Table 5 shows the relationship between LV and normal liver in patients with liver disease separated by gender. The LV of females with cirrhosis was significantly smaller than that of females with normal liver (P = 0.048).

**Table 3:** Correlation between liver volume and biochemical blood test data in normal and diseased liver groups of males.

Males	Normal liver (n = 137)		Diseased liver (n = 55)	
	R	P value	R	P value
Albumin	0.055	0.528	0.265	0.051
Bilirubin	0.044	0.613	0.385	0.004
AST	0.060	0.487	0.201	0.142
ALT	0.030	0.731	0.064	0.644
PLT	0.123	0.158	0.144	0.294

AST: aspartate transaminase, ALT: alanine transaminase, PLT: platelet.

**Table 4:** Correlation between liver volume and biochemical blood test data in normal and diseased liver groups of females.

Females	Normal liver (n = 138)		Diseased liver (n = 44)	
	R	P value	R	P value
Albumin	0.080	0.350	0.304	0.045
Bilirubin	0.178	0.037	0.092	0.551
AST	0.083	0.332	0.279	0.066
ALT	0.030	0.725	0.129	0.404
PLT	0.389	< 0.0001	0.312	0.039

AST: aspartate transaminase, ALT: alanine transaminase, PLT: platelet.

**Table 5:** Relationship between liver volume and normal liver in patients with liver disease separated by gender.

Males	n	Liver volume	P	Females	n	Liver volume	P
Normal liver	137	1188.64	-	Normal liver	138	969.45	-
		[974.36–1344.91]				[848.25–1132.28]	
Chronic hepatitis	26	1205.36	0.526	Chronic hepatitis	26	944.81	0.787
		[1064.70–1341.97]				[805.91–1139.23]	
Liver cirrhosis	4	1118.39	0.798	Liver cirrhosis	7	697.74	0.048
		[1048.44–1343.17]				[551.91–1198.54]	
HCC	5	1177.26	0.335	HCC	1	906.3	0.680
		[1051.61–1713.97]					
Bile duct disease	20	1230.81	0.085	Bile duct disease	10	981.09	0.591
		[1085.31–1386.46]				[792.94–1105.39]	

HCC: hepatocellular carcinoma.

We found a clear relationship between LV, gender, and age from 374 consecutive MDCT volumetry measures. LV in the males was significantly higher than in the females. Furthermore, LV was significantly correlated with BSA rather than with BMI and negatively correlated with age at the same time. Finally, we found that LV was correlated with age in the normal liver but not in the diseased liver.

We showed a large difference in LV between males and females. The LV in the males became smaller than the LV in the females, along with aging. It could be due to hormonal differences between the genders [30]. Estrogen was found to be a hepatotropic factor, which could facilitate liver regeneration [31]. The role of estrogen in the liver in aging was previously unclear. However, it could play an important role in maintaining LV in the female, along with aging. Liver disease induces feminization in male patients due to deterioration of the estrogen metabolism [32]. Therefore, LV in the diseased male liver was maintained in elderly patients due to the elevation of estrogen levels.

LV showed a good correlation with BSA rather than with BMI. Yoshizumi et al. have shown that BSA is more correlated than BMI using deceased liver donors [22]. Vauthey et al. also conclude that BMI did not have an independent effect on LV [33]. The exact reasons for the difference between BSA and BMI to LV were unclear. It could be a mathematical issue associated with the formula. However, BSA could be correlated with visceral volume, whereas visceral volume could not be associated with BMI, which stands for visceral fat deposition. Therefore, LV would be more associated with height than with weight. Questions for further consideration are which formula is suitable to calculate real BSA and how a novel method may be invented to estimate BSA.

LV is correlated with age in the normal adult liver, but not in the diseased liver. Height is likely unchanged throughout life, however, weight may change due to loss of basic metabolism [34] and advancing frailty [35]. Therefore, BSA would be unstable throughout life. Thus, we need to modify LV after adjusting constant BSA. We clearly showed that LV would decrease with aging. It is unclear if a decrease in LV may produce a decrease in liver function. As shown in Tables 3 and 4, albumin and bilirubin may correlate with LV in the case of diseased liver in males, whereas platelets correlate with LV independently of background in females, although bilirubin was not significantly associated. As shown in Table 5, the LV in cases of females with cirrhosis tended to decrease, but no change was observed in males. Thus, it is possible to predict liver atrophy due to hormones and other factors in females. The fact that platelets correlate with LV regardless of background in females and that atrophy occurs in patients with cirrhosis suggests a stronger correlation between LV and liver function in females. In contrast, it may be more difficult to evaluate potential condition in males due to the discrepancy between LV and liver function. Given the differences between males and females in terms of LV and liver function, evaluations should be separated by gender. The LV in the diseased liver would be atrophic and reduced in size and weight in the acute phase of illness, and LV would be maintained in the chronic phase. Therefore, surgeons should consider both the liver function associated with liver disease and the age-related loss of LV when clinically planning hepatectomy.

Several criteria, such as Barcelona criteria [36], Makuuchi criteria [4], Sapporo criteria [37], and Hongkong criteria [38], have been established for liver resection. Furthermore, Vauthey et al. reported a consensus statement on the evaluation of hepatocellular carcinoma [39]. The consensus statement recommended that portal vein embolization be considered if the residual LV is low [39]. The volume for portal vein embolization depends on liver function, such as at least 20% in the normal liver, at least 30% in the injured liver, and at least 40% in the compensatory fibrous or cirrhotic liver. The statement represents clear relation between LV and its function depending on disease progression. As shown in this study, the aging effect should be included in future criteria for liver resection.

There are several limitations of this study. First, we did not recruit a normal patient population. Therefore, we are not sure if the normal liver in this study would represent a normal population. Second, we did not follow the patients, and therefore, all the data are independent. We cannot exclude the possibility that any alteration would be due to the generation effect. Third, the degree, type, and stage of disease in affected livers were unknown. To exclude all bias, a large prospective trial is warranted.

## Conclusion

We investigated the changes in LV between the genders along with aging. In conclusion, LV in the normal liver showed a negative correlation with age, and LV in the males was more correlated to aging than LV in the females. This study indicates that the aging effect on LV should be considered in future protocols for liver resection.

## Supporting information

S1 File (XLSX)
